# *micro*RNA and Metabolite Signatures Linked to Early Consequences of Lethal Radiation

**DOI:** 10.1038/s41598-020-62255-w

**Published:** 2020-03-25

**Authors:** Nabarun Chakraborty, Aarti Gautam, Gregory P. Holmes-Hampton, Vidya P. Kumar, Shukla Biswas, Raina Kumar, Dana Hamad, George Dimitrov, Ayodele O. Olabisi, Rasha Hammamieh, Sanchita P. Ghosh

**Affiliations:** 1 The Geneva Foundation, Medical Readiness Systems Biology, Walter Reed Army Institute of Research, Fort Detrick, MD 21702-5010 USA; 2 Medical Readiness Systems Biology, Walter Reed Army Institute of Research, Fort Detrick, MD 21702-5010 USA; 3 ORISE, Medical Readiness Systems Biology, Walter Reed Army Institute of Research, Fort Detrick, MD 21702-5010 USA; 4 Armed Forces Radiobiology Research Institute, Uniformed Services University of the Health Sciences, Bethesda, MD 20889 USA

**Keywords:** Biotechnology, Computational biology and bioinformatics, Immunology, Systems biology, Health care, Signs and symptoms

## Abstract

Lethal total body irradiation (TBI) triggers multifactorial health issues in a potentially short time frame. Hence, early signatures of TBI would be of great clinical value. Our study aimed to interrogate *micro*RNA (miRNA) and metabolites, two biomolecules available in blood serum, in order to comprehend the immediate impacts of TBI. Mice were exposed to a lethal dose (9.75 Gy) of Cobalt-60 gamma radiation and euthanized at four time points, namely, days 1, 3, 7 and 9 post-TBI. Serum miRNA libraries were sequenced using the Illumina small RNA sequencing protocol, and metabolites were screened using a mass spectrometer. The degree of early impacts of irradiation was underscored by the large number of miRNAs and metabolites that became significantly expressed during the Early phase (day 0 and 1 post-TBI). Radiation-induced inflammatory markers for bone marrow aplasia and pro-sepsis markers showed early elevation with longitudinal increment. Functional analysis integrating miRNA-protein-metabolites revealed inflammation as the overarching host response to lethal TBI. Early activation of the network linked to the synthesis of reactive oxygen species was associated with the escalated regulation of the fatty acid metabolism network. In conclusion, we assembled a list of time-informed critical markers and mechanisms of significant translational potential in the context of a radiation exposure event.

## Introduction

In the current geopolitical climate, the threat of radiation exposure either through intentional detonation or accidental exposure represents an ever present concern. To this end, it is imperative that we bolster our understanding of the systems and biological processes that are affected by exposure to ionizing radiation. Collectively the symptoms and health consequences that follow radiation exposure are referred to as acute radiation syndrome (ARS). It has been established that exposure to different levels of radiation can lead to different sets of sub-syndromes (i.e., hematopoietic, gastro-intestinal [GI], and central nervous system [CNS])^1^. Each of these is associated with the severity of the absorbed dose of radiation; bone marrow is regarded as the most susceptible tissue to radiation exposure, and the hematopoietic sub-syndrome occurs at the lowest doses of radiation. Many medicinal countermeasures are targeted at doses of radiation in the hematopoietic range. At higher doses of radiation, there can be damage to the gastro-intestinal system (GI). At very high doses, organisms can be susceptible to damage in the CNS leading to the rapid onset of symptoms that often result in mortality.

In the mouse, the range for hematopoietic ARS is generally accepted to be from 2 to 10 Gray (Gy)^2,3^; however, even amongst various strains of mice, the radiation dose for lethality can vary. For instance, in a recent study investigating the medical countermeasure Filgrastim^4^, four mouse strains were studied, and four different radiation doses for 50% lethality at 30 days (Lethal Dose or LD50/30) were identified. For the inbred strains such as C3H/HeN and C57BL/6, the doses for LD50/30 were identified as 7.56 and 7.74 Gy, respectively. In the B6C3F1 strain, which is a cross between the two inbred strains, the value for LD50/30 was identified as 8.12 Gy, and in the CD2F1 strain, which is a cross between the BALB/c and DBA/2, the LD50/30 value is even higher at 8.65 Gy. Furthermore, the sex of the mouse changes the response to radiation exposure^5^. Mice experiencing hematopoietic ARS often exhibit decreased numbers of peripheral blood cells, leading to neutropenia and thrombocytopenia, which can result in death often from infection or bleeding.

At higher radiation doses in the mouse (~10 Gy and above), damage resulting from radiation exposure can be observed in the GI system in addition to bone marrow, and weight loss; and diarrhea are typically observed, which can lead to dehydration and mortality^6^. There is an increasing traction about determining the role of serum miRNAs in responding to radiation, particularly in the context of biodosimetry^7,8^. A number of reviews highlighted the potential utility of miRNAs as predictors of carcinoma prognosis^9,10^ and of drug efficacy^11,12^. The direct relationship of miRNA and radiation exposure was further underscored by a study demonstrating differential expression of miRNAs in C57BL/6 mice exposed to either a sub-lethal dose of 6.5 Gy or a lethal dose of 8 Gy, and in which, the levels of miRNA perturbation reverted back to baseline after administration of amifostine as a medical countermeasure^13^. In this study, authors identified several differentially expressed miRNAs, such as miR-126-3p, miR-150, miR-342-3p, miR-151-3p, miR-139-3p, and miR-142 and saw overlap with results from previous studies^7,14,15^. In an earlier report (2016)^16^, we have shown that radiation-induced miRNAs in the spleen might be involved in radioprotection by a vitamin E analog, gamma-tocotrienol (GT3). Functional analysis indicated that the radiation-induced miRNAs, which were modulated by GT3 were involved in hematopoiesis and lymphogenesis^16^. The radiation dose and time lag following exposure are two critical factors that essentially define the degree of consequences of radiation. For instance, the hematopoietic symptoms of ARS typically takes a certain period of time to develop *in vivo*, as the mortality begins weeks after radiation exposure^1^. By contrast, animals that have been treated with a radiation dose high enough to induce the GI sub-syndrome will typically exhibit symptoms within the first several days, and mortality begins in less than a week^1^. For the purpose of our present study, we functionally defined the Early period relative to radiation exposure as the first two days post-TBI. Likewise, we defined the Late period as the ninth day after irradiation. The interim period was defined as the Mid period. Bridging the knowledge gap about the temporal impact of radiation exposure^17,18^; here we present a longitudinal pattern of miRNA shift promoted by lethal dose of radiation.

Another emerging field is the study of metabolomics in biosamples collected from animals that have been exposed to radiation^19^. Metabolomics is a powerful tool to study post-radiation changes in metabolism and bioenergetics even before the onset of clinical symptoms and can augment triage of an exposed population in real-life scenarios^19,20^. Past metabolomics analysis investigated mouse^21^ and rat^22^ urine, rat plasma^23^, and mouse livers^24,25^. In 2013, we reported for the first time radiation-induced metabolite changes in the GI tissues of mice using ultraperformance liquid chromatography (UPLC) coupled with Time-of-flight mass spectrometry (TOF-MS)^20^. Mice were exposed to TBI doses of 4 and 8 Gy, and blood and jejuna were collected at 1 and 4 days post-irradiation^20^. Novel metabolites including lipids, glutamate, tryptophan, taurocholate and the Cysteine-Glycine dipeptide were identified^20^. In addition, human serum samples have been analyzed in individuals following radiotherapy^26,27^; these studies indicated upregulation of biomarkers corresponding to energy metabolism including increased branched chain amino acid synthesis and protein biosynthesis^27^. C57BL/6 mouse biosamples including bone marrow, ileum, muscle, lung, serum, and urine have been analyzed within 12 hours of 6 Gy irradiation and demonstrated differences in DNA methylation pattern, energy expenditure and metabolism of amino acid, glutathione, and bile, respectively^28^. Unlike miRNA profiling, radiation-induced metabolite profiles at various time points relative to radiation exposure warrants further study. In the current study, we aimed to comprehend the longitudinal dynamics of serum miRNA-metabolite landscape perturbed by lethal radiation. For these studies, we used CD2F1 male mice that were exposed to TBI at a GI sub-syndrome-inducing level of 9.75 Gy. These mice were ~12 weeks old at the time of irradiation which is equivalent to the age of a young adult when comparing the mouse lifespan of a mouse to a human^29^. Serum samples were collected in Early, Mid, and Late time points relative to TBI. Our result identified a set of miRNA and metabolite markers. The networks co-enriched by differentially expressed miRNA and metabolites suggested a surge of inflammatory response to lethal radiation. A number of disease footprints related to hematopoiesis and cardiovascular atrophy emerged as the potential targets for immediate intervention.

## Materials and Methods

### Handling of mice

Mouse were sourced and housed as described previously^30^. Briefly, CD2F1 male mice (8–10 weeks old) were purchased from Harlan (Indianapolis, IN) and housed in an air-conditioned facility at the Armed Forces Radiobiology Research Institute (AFRRI) which is accredited by the Association for Assessment and Accreditation of Laboratory Animal Care International (AAALAC). All mice were kept in rooms with a 12 h light/dark cycle at 21 ± 2 °C with 10–15 hourly cycles of fresh air and a relative humidity of 50 ± 10%. Mice were held in quarantine for 2 weeks and were used after microbiology, serology, and histopathology examination of representative samples ensured the absence of *Pseudomonas aeruginosa* and common murine diseases. Mice were given certified rodent rations (Harlan Teklad Rodent Diet #8604, Harlan Teklad, WI, USA) and acidified water (with HCl, pH 2.5–3.0) *ad libitum*. Animal procedures were executed following a protocol approved by the AFRRI’s Institutional Animal Care and Use Committee (IACUC). Research was conducted according to the Guide for the Care and Use of Laboratory Animals, prepared by the Institute of Laboratory Animal Resources, the National Research Council, U.S. National Academy of Sciences^16^.

### Irradiation

Mice were irradiated bilaterally in the AFRRI cobalt-60 gamma radiation facility in well ventilated plexiglass boxes (8 mice per box) at a dose rate of ~0.6 Gy/minute (min) to total midline doses of 9.75 Gy, as described below. An alanine/electron spin resonance (ESR) dosimetry system (American Society for Testing and Material Standard E 1607) was used to measure dose rates (to water) in the cores of acrylic mouse phantoms. Phantoms were 3 inches long and 1 inch in diameter and were located in 50% of the compartments of the exposure rack. The ESR signals were measured with a calibration curve based on standard calibration dosimeters provided by the National Institute of Standard and Technology (NIST, Gaithersburg, MD). The accuracy of the calibration curve was verified by inter-comparison with the National Physical Laboratory (NPL) in the United Kingdom. The only corrections applied to the dose rates in phantoms were for the decay of the ^60^Co source and for a small difference in mass energy-absorption coefficients for water and soft tissue. The radiation field was uniform within ±2%. After irradiation, mice were returned to their original cages with access to food and water *ad libitum*^17,30^. All irradiations were performed in the morning to minimize the diurnal effect.

### Blood collection

The experimental animals received either 0 or 9.75 Gy radiation (lethal dose) at a dose rate ~0.6 Gy min^−1^ in the AFRRI ^60^Co gamma radiation facility. Blood was collected from inferior vena cava under anesthesia on days 0 (2 h post-TBI), 1, 3, 7, and 9 from either 9.75 Gy-irradiated or unirradiated mice followed by euthanasia. Serum was separated and stored at −80 °C until use.

### *micro*RNA sequencing and analysis

We followed the protocol reported by us elsewhere with necessary modification^31^. Briefly, 5 µL serum was used to construct sequencing libraries with the Illumina (Illumina, CA, USA) TruSeq Small RNA Sample Prep Kit, following the manufacturer’s guideline. Briefly, 3′ and 5′ adapters were sequentially ligated to small RNA molecules, and the ligated products were reverse transcribed, amplified and subsequently size-selected screened by gel purification and by microfluidics using the Agilent Bioanalyzer High Sensitivity DNA chip. The indexed libraries were pooled in equimolar amounts, clustered and loaded into different lanes of a HiScanSQ Illumina instrument to generate 50 base-pair reads. Image analysis and base calling were performed using Illumina pipeline Version 1.5.15.1 and Illumina CASAVA sequencing analysis software Version 1.7.32.0. The raw sequence reads were processed using a robust miRNA-seq quality control pipeline. First, the samples were evaluated as per quality control assurance using matrices that included acceptable duplication, k-mer or GC content generated using FASTQC. The samples that showed more than 30% disagreement with rest of the samples were excluded henceforth. The low quality reads (Q20 quality score threshold) were filtered out, and adaptor sequences were pruned. The ensuing products were 48%–75% mapped against the mature miRBase miRNA database Version 21 for Mus musculus (mm9), a pool of RNA species was curated, and the effective library sizes were normalized using the trimmed mean of M-values (TMM) normalization method provided by edgeR (Bioconductor.org). In a second phase of filtration, the samples with <1% abundance were also discarded. Meanwhile, we validated the miRBase output by mapping the same filtered sequence reads against the UCSC reference genome for Mus musculus (mm9 build) (University of California Santa Cruz; http://genome.ucsc.edu/).

The short read aligner Bowtie (v 1.0.0) allowed one pair of mismatch. For the baseline, we combined the miRNA readouts obtained from the serum samples collected from two control time points (C1 and C7). Differentially expressed miRNAs were mined by pair-wise comparison between the baseline and individual time points using a moderated t test with the cutoff at *p* < 0.05.

### Protein ELISA assay

Mouse Erythropoietin (EPO) Quantikine ELISA and mouse/Rat Flt3 ligand (Flt3L) quantikine ELISA kits were purchased from R&D Systems Inc. (Minneapolis, MN). Mouse Serum Amyloid A (SAA) quantikine ELISA kits were purchased from Tridelta Development Limited (Maynooth, County Kildare, Ireland). The cytokine detection limits were 18 pg mL^−1^, >5 pg mL^−1^ and 30 ng mL^−1^ for EPO, Flt-3L and SAA ELISAs, respectively. The quantitative levels of EPO, Flt3L, and SAA were evaluated from serum samples collected on days 1, 3, 7 and 9 post-TBI following standard protocols from the vendor. The ELISA result in pg/mL was first computed for individual pre-irradiation baseline and post-irradiation time points, respectively. Fold change was calculated by dividing the irradiation data points by the pre-irradiation baseline data. One-way ANOVA followed by multiple comparison was computed to find the significant changes.

### Global metabolite mass spectroscopy assay

The metabolite profiling of serum samples was conducted as per the report published previously^32^. Here each sample (5 μL) was injected onto a reverse-phase 50 × 2.1 mm Acquity 1.7-μm C18 column (Waters Corp, Milford, MA) using an Acquity UPLC system (Waters) with a gradient mobile phase consisting of 2% acetonitrile in water containing 0.1% formic acid (Solvent A) and 2% water in acetonitrile containing 0.1% formic acid (Solvent B) and resolved for 10 min at a flow rate of 0.5 mL/min. The gradient was consisted of 100% A for 0.5 min with a ramp of curve 6–100% B from 0.5 to 10 min. The column eluent was introduced directly into the mass spectrometer by electrospray. Mass spectrometry was performed on a Quadrupole Time-of-Flight (Q-TOF) Premier mass spectrometer (Waters) operating in either positive-ion or negative-ion electrospray ionization (ESI+/−) mode with a capillary voltage of 3200 V and a sampling cone voltage of 20 V in negative mode and 35 V in positive mode. The desolvation gas flow was set to 800 L/hour, and the temperature was set to 350 °C. The cone gas flow was 25 L/hour, and the source temperature was 120 °C. Accurate mass was maintained by introduction of LockSpray interface of sulfadimethoxine (311.0814 [M + H]+ or 309.0658 [M − H]−) at a concentration of 250 pg/μL in 50% aqueous acetonitrile and a rate of 150 μL/min. Data were acquired in centroid mode from 50 to 850 mass to change ratio (m/z) in mass spectrometry scanning. Centroided and integrated mass spectrometry data obtained in duplicates from the UPLC-TOF mass spectrometer were processed using XCMS (Scripps Institute) to generate a data matrix containing ion intensities, m/z and retention time values. Peaks were machine normalized and mined with ppm error cutoff <1. Baseline was defined as the serum samples collected at two control cohorts (C1 and C7). Significantly different peaks at individual time points were selected using moderated t-test *p* < 0.05.

### Statistical analysis

GeneSpring 14.9 (Agilent Technologies, Inc., Santa Clara, CA) was used to find statistically significant markers and to compute principal component analysis (PCA) and clustering analysis. K-means clustering was performed using Euclidian similarity measures and 50 iterations. Hierarchical clustering was conducted using Similarity Measure: Squared Euclidean similarity measures and Wards linkage rule. Prism 7 (GraphPad, Inc. San Diego, CA) was used for data visualization.

The differentially expressed mass spectroscopy peaks were annotated using the CEU Mass Mediator 3.0 database (www.ceumass.eps.uspceu.es), and the molecules were screened based on the following guidelines: (a) Ppm error less than 1; (b) chemical formula comprised of the adducts +H, −H, +Na, +K, +NH4, −Cl and (c) chemicals listed as endogenous mammalian.

Ingenuity Pathway Analysis (IPA, QIAGEN, Inc., version 01–13) was used for functional analysis and network building. Those miRNAs and metabolites, which were differentially expressed at each phases post-TBI, that is Early-, Mid- and Late-phase post-TBI, were seeded in the IPA analysis portal; and the canonical and non-canonical networks significantly enriched (hypergeometric test, *p* < 0.05) at each phase were curated. We further screened the list to select those significantly enriched networks, which were enriched by at least 5 molecules that included miRNA and/or metabolites. Finally, we used Venn diagram to screen the networks which were significantly enriched across the time phases post-TBI.

## Results

**Figure 1:**
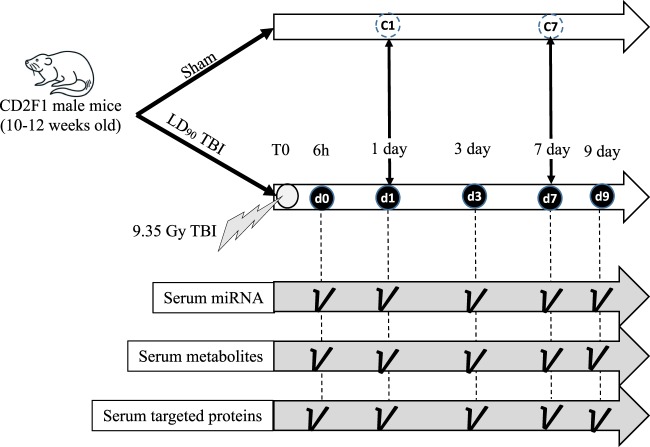
Study design. CD2F1 male mice were exposed to LD90 total body irradiation (TBI). Post-TBI, mice were euthanized at five time points: 6 h (marked as d0), one day (d1), three days (d3), seven days (d7) and nine days (d9). Two control cohorts (C1 and C7) accounted for the baseline. Whole genome miRNA profiling, global metabolomics and targeted metabolomics were conducted at each time point.

The mice were exposed to 9.75 Gy TBI. Post-irradiation, the mice were longitudinally euthanized at five different time points. One cohort was euthanized two hours after the irradiation, and the time point was defined as day 0 (d0). The post-TBI time points included day one (d1), day three (d3), day seven (d7) and day nine (d9). Sham animals were euthanized at two different time points to take into account the aging factor: day one (C1) and day seven (C7), which were equivalent to d1 and d7 post-TBI. All the cohorts including both irradiated and sham consisted of five mice per group (Fig. 1). At this radiation dose (9.75 Gy) CD2F1 male mice (12–14 weeks old) typically exhibit clinical symptoms from days 9 to 10 post-TBI^18^. Clinical symptoms include hunched posture, difficulty in breathing and slow movement^33^. About 90–95% mortality has been observed over 30 days at this dose^18,30^.

### miRNA analysis

**Figure 2:**
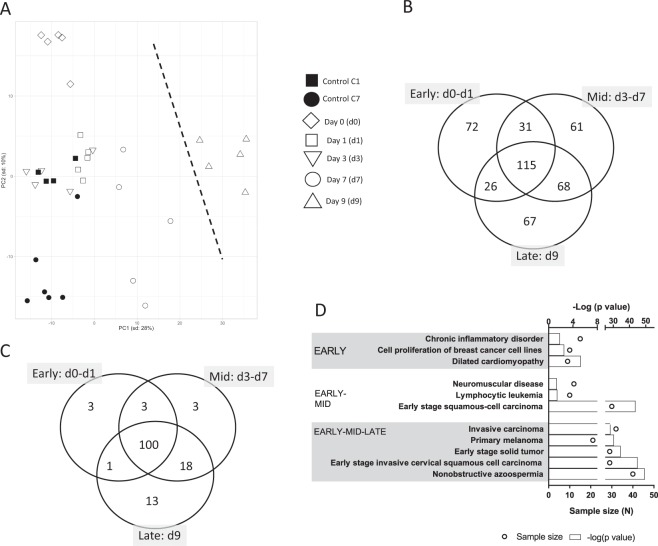
The whole genome miRNA profile responding to lethal radiation. (**A**) Principal component analysis (PCA) of the whole genome miRNA data shows a clear separation between d9 post-TBI and rest of the time points including the controls. A dotted line marks the separation. (**B**) Venn diagram shows the longitudinal distribution of differentially expressed miRNAs across three time phases post-TBI, namely, Early (d0–d1), Mid (d3–d7) and Late (d9). The largest number of differentially expressed miRNAs (115 miRNAs) are found at the cross section of the three phase. Nevertheless, a considerable number of miRNAs are exclusively regulated during each of the three phases. For instance, there are 72, 61 and 67 miRNAs exclusively regulated in the Early, Mid and Late phases, respectively. (**C**) Venn diagram shows the longitudinal distribution of significantly enriched networks across three time phases post-TBI, namely, Early (d0–d1), Mid (d3–d7) and Late (d9). Nearly all of these networks are concentrated at the cross section of all three time phases. (**D**) Top 5 most significantly enriched networks during the Early, Early-Mid and Early-Mid-Late phases are shown in this bar chart. Early and Early-Mid phases enlist only 3 networks, so all of them are included here. Top horizontal axis represents the −log(p value), and the bar charts represents the hypergeometric p-values converted to log_10_ associated with individual networks. The bottom horizontal axis represents the sample size (N), and the open circles represent the number of miRNAs associated with individual networks.

PCA analysis of serum miRNA of individual mice was plotted in Fig. 2A. The first two principal components explained 31.2% of total variance with 28% attributable to PC1. Baseline controls (C1 and C7) were clustered together with early irradiation time points (d0 and d1). Mice euthanized on d7 post-irradiation were clustered separately, and mice euthanized at d9 were clustered far from the rest the mice across PC1.

Based on the time course and the clustering trend in PCA, we grouped the mice in three temporal phases. Early phase included those that were euthanized at d0 and d1 time points. Table 1 lists the top 5 over-expressed and top 5 under-expressed miRNAs and their relevant functions associated with the Early phase post-TBI. Mid phase included d3 and d7 time points. The Late phase had a single entry of d9 time point.

**Table 1:** List of top 5 highly upregulated and downregulated microRNAs during Early phase post-TBI.

miRNA	Log_2_(Fold Change)	Relevance to present objective
miR-714	5.19	Significantly upregulated in rodent thymic lymphoma cells at six months post-TBI^74^
miR-3971	5.02	Stimulates cellular production
miR-150-5p	4.80	Cross-species validated radiation marker for time- and dose-dependent reduction in expression in plasma^8^. We found subsequent reduction in expressions in the Mid (−1.31) and Late phases (−1.53).
miR-5120	4.76	Decreased in hematopoietic progenitor cells exposed to pollutants, like benzene^75^
miR-5104	4.63	Another candidate to be decreased in hematopoietic progenitor cells exposed to pollutants, like benzene^75^
miR-204-5p	−5.24	Reduced serum load, an established oncogenic marker^76^
miR-96-5p	−6.14	Oncogenic marker and apoptosis regulator^77^
miR-124-3p	−6.25	Decreased load promotes cancer^78^
miR-9-5p	−6.57	Reduced expression as marker of bone-related cancer^79^
miR-211-5p	−7.21	Overexpressed miR-211-5p and miR-204-5p promote resistance against melanoma^80^

A Venn diagram (Fig. 2B) curated 72 miRNAs, which were exclusively regulated during the Early phase post-TBI. This Early-phase exclusive group included 57 over-expressed and 15 under- expressed miRNAs. In addition, 31 miRNAs were concurrently expressed in the Early and Mid phases. This set included 6 and 10 miRNAs consistently over expressed and under expressed in both time phases; the remaining 15 miRNAs altered their regulations between the Early and Mid phases. There were 115 miRNAs conserved among all three post-TBI phases that included 51 consistently over-expressed and 42 consistently under-expressed miRNAs. Figure S1 showed a hierarchical clustering of these 115 miRNAs conserved across the entire post-TBI phases. Table S1 lists the entire set of differentially expressed miRNAs sorted based on their temporal distribution as noted in the Venn diagram.

### Biological networks

For functional analysis, we curated the biological networks differentially enriched by the three sets of miRNA linked to the respective temporal phase post-TBI. The Venn diagram (Fig. 2C) displayed the temporal distribution of those networks (see Table S2 for complete list of networks). The networks linked to cardiomyopathy, cell proliferation, and inflammation were exclusively enriched during the Early phase (Fig. 2D). It is important to note that 109 out of 144 networks (i.e., nearly 75% of all networks enriched by three time phases combined) were significantly enriched during the Early phase. Furthermore, 100 out of the 109 networks (i.e., nearly 91% of all networks enriched by three time phases combined) were co-enriched by all three time phases. Analyzing further, we noted 23 networks significantly enriched by over-expressed miRNAs during the Early phase post-TBI, and 12 of these 23 networks were related to abnormal cell growth. In fact, a substantial number of tumorigenesis and carcinogenesis networks were significantly enriched across all three time phases; Fig. 2D lists those networks that were associated with tumorigenesis and carcinogenesis, and emerged significantly enriched starting from the Early phase post-TBI.

### Inflammatory response after radiation injury

**Figure 3:**
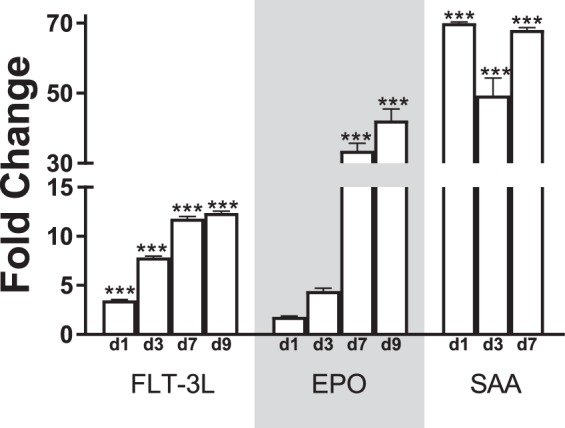
Expression of FLT-3L, EPO, and SAA from ELISA measurement. The longitudinal expression of FLT-3L, EPO, and SAA is plotted in the bar chart. The fold changes are calculated from the baseline control (C0). ***p < 0.001; N = 10 for each time point.

To demonstrate the radiation-induced damage in the irradiated animals exposed to 9.75 Gy of TBI, we tested the markers of bone marrow aplasia (EPO and FLT-3L) and sepsis (SAA) in serum at different times post-radiation (Fig. 3). Starting from the d1 post-TBI, FLT-3L levels were significantly elevated compared to the non-irradiated control (*p* < 0.001), and the trend continued through d9 post-TBI. A linear regression model found a significantly increasing trend in the protein expression dynamics across this time point (slope = 2385 ± 282; F = 71.6; DF_n_ = 1; DF_d_ = 3, *p* = 0.003). On the other hand, the level of EPO was significantly elevated (*p* < 0.001) from d7 post-TBI and onwards. Yet again, the linear regression model demonstrated an increasing load of EPO across these time points (slope = 2085 ± 528; F = 15.6; DF_n_ = 1; DF_d_ = 3, *p* = 0.03). A very high fold change was registered in SAA starting from day 1 (Fold change: 70 ± 35; *p* < 0.001) and remained highly upregulated through d7 post-TBI.

### Metabolomic analysis

Table S3 lists all the peaks identified by the mass spectrometer. Henceforth, we used a subset of these peaks, which were annotated by Human Metabolite Database (HMDB) and differentially expressed from the baseline (see Table S4 for entire metabolite set). Table 2 lists the top 5 over-expressed and top 5 under-expressed metabolites found in the Early phase, and their biofunctions relevant to present study.

**Table 2:** List of top 5 highly upregulated and downregulated metabolites during Early phase post-TBI.

Metabolite	Log_2_(Fold Change)	Relevance to present objective
Diacylglycerol	0.79	Linked to fatty acid synthesis in cancer cells^81^ and insulin resistance^82^
Hydrocortisone	0.79	Higher hydrocortisone correlates with and regulates cardiac function^83^
Phosphatidylcholine	0.55	A marker of oxidative stress^84^
Cholesteryl oleate	0.53	Linked to cardiac dysfunction^85^
Triacylglycerol (isomer 6)	0.52	A monopalmitic acid triglyceride
Phosphatidylethanolamine	−0.60	Along with phosphatidylcholine, phosphatidylethanolamine plays a significant role in mitochondrial function, activities several enzymes
Stearylamine	−0.65	Immuno-modulator^86^
Phosphatidylcholine	−0.67	Along with phosphatidylethanolamine, phosphatidylcholine plays significant role in mitochondrial function
Docosapentaenoic acid	−0.79	An omega-3 fatty acid with multiple health benefits
Triamterene	−0.98	Controls nephrotoxicity
Allyl sulfide	−1.62	Facilitates anticancer activities^87^

**Figure 4:**
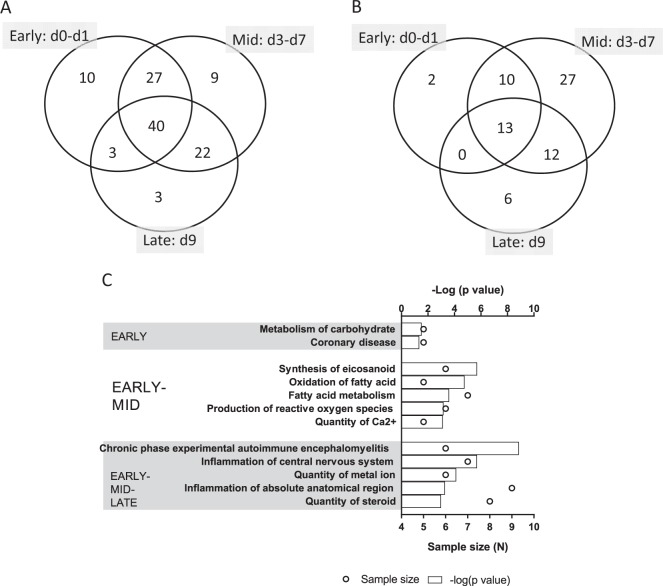
The global metabolite profile responding to lethal radiation. (**A**) Venn diagram shows the longitudinal distribution of differentially expressed metabolites across three time phases post-TBI, namely, Early (d0–d1), Mid (d3–d7) and Late (d9). The largest number of differentially expressed metabolites (40 metabolites) is found at the cross section of the three phases. (**B**) Venn diagram shows the longitudinal distribution of significantly enriched networks across three time phases post-TBI, namely, Early (d0–d1), Mid (d3–d7) and Late (d9). Unlike the miRNA profile, here most of the networks are linked to the Mid phase post-TBI. (**C**) The top 5 most significantly enriched networks during the Early, Early-Mid and Early-Mid-Late phases are shown in this bar chart. The Early phase enlists only 2 networks, so all of them are included here. The top horizontal axis represents the −log(p value), and the bars represent the hypergeometric p-values converted to log_10_ associated with individual networks. The bottom horizontal axis represents the sample size (N), and the open circles represent the number of metabolites associated with individual networks.

A Venn diagram (Fig. 4A) showed that 10 metabolites were exclusively expressed in the Early phase, which included 4 over-expressed and 6 under-expressed metabolites. There were 27 metabolites co-regulated in the Early and Mid-phases, of which 6 and 16 metabolites were consistently over expressed and under expressed, respectively, in the Early and Mid phases post-TBI. The remaining 5 metabolites changed their regulations between the Early and Mid phases. In addition, 40 metabolites (Fig. S2) were co-regulated in all three post-irradiation phases, including 25 consistently over expressed and 11 consistently under expressed metabolites among the Early, Mid and Late phases post-TBI.

### Network analysis

The functional analysis was performed for the differentially expressed HMDB-annotated metabolites expressed in the Early, Mid and Late phases post-TBI. The Venn diagram (Fig. 4B) depicted the temporal trend of the networks significantly enriched in all three time phases (Table S4 enlists all networks). Figure 4C shows the most significant networks potentially relevant to early markers of TBI. Unlike the miRNA-enriched networks, a majority of the metabolite-enriched networks (62 out of 70) were discovered to be enriched during the Mid phase post-TBI. Interestingly, seven out of ten networks co-enriched during the Early and Mid phases post-TBI were associated with metabolism. These metabolism-associated networks included oxidation of fatty acids (*p*-values at Early phase: 1.74 × 10^−5^, and Mid phase: 9.49 × 10^−7^), concentration of triglyceride (*p*-values at Early phase: 3.45 × 10^−3^, and Mid phase: 3.87 × 10^−5^), fatty acid metabolism (*p*-values at Early phase: 2.47 × 10^−4^, and Mid phase: 2.14 × 10^−4^), concentration of lipid (*p*-values at Early phase: 1.22 × 10^−3^, and Mid phase: 3.04 × 10^−4^) and concentration of cholesterol (*p*-values at Early phase: 2.78 × 10^−3^, and Mid phase: 2.53 × 10^−5^). The network linked to blood pressure was consistently enriched during the Early (*p* = 2.70 × 10^−3^) and Mid phases (*p* = 2.80 × 10^−3^) post-TBI. In concert, a network linked to coronary disease was significantly perturbed in the Early phase (*p* = 0.045).

Finally, 13 networks were consistently perturbed across the 3 time phases post-TBI, of which 8 were linked to reactive oxygen species (ROS) signaling and immune functions. Figure S3 depicts the network linked to ROS synthesis and causally related to metabolic functions, such as glucose and fatty acid metabolism. This non-canonical network was comprised of 3 upregulated and 9 downregulated metabolites.

**Figure 5:**
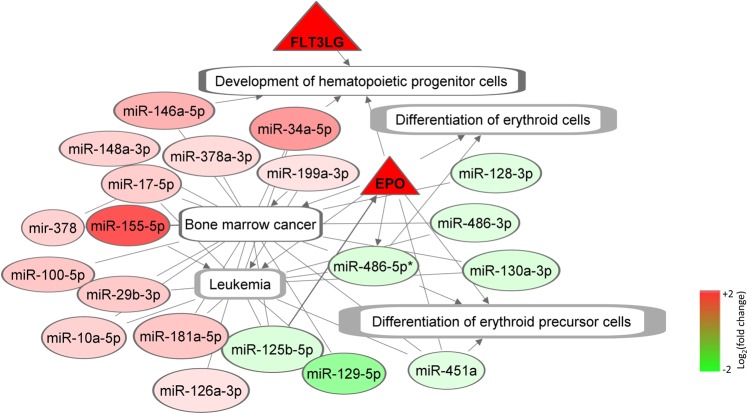
The non-canonical network linked to hematopoiesis. This network was generated around the two hub proteins FLT-3LG and EPO. Both of these proteins were upregulated during the Early phase post-TBI. FLT-3LG was significantly (*p* < 0.001) elevated from baseline. EPO was not significantly elevated during the Early phase, but its expression gradually increased to meet the significance level at d7 post-TBI. In this network, these proteins are marked by triangular nodes. The miRNAs enriching hematopoiesis networks are marked by the oval-shaped nodes. Furthermore, we identified five significantly enrihed (*p* < 0.05) sub-networks under the overarching hematopoiesis network, namely development of hematopoietic progenitor cells, differentiation of erythroid cells, differentiation of erythroid precursor cells, bone marrow cancer and leukemia. The nodes representing these biological functions and dieases are rectangular in shapes. The arrow-headed and blunt-headed edges represent activating and associative relationship between two nodes, respectively. All of the protein and miRNA nodes are colored based on their regulation levels observed during the Early phase post-TBI, and the color code is at right bottom. The rectangular nodes of the subnetworks representing the biological functions and diseases remained uncolored.

Integrating the proteins and miRNA, a non-canonical hematopoiesis network was generated using FLT-3L and EPO as the two-hub molecule (Fig. 5). In addition to these two proteins, this network consists of 20 miRNAs differentially expressed at the Early phase. Relevant non-canonical networks enriched by miRNAs included bone marrow cancer (*p* = 0.0038), hematologic cancer (*p* = 1.2 × 10^−5^) and leukopoiesis (*p* = 0.02).

Cardiovascular disorder emerged as one of the major disease footprints co-enriched by miRNA and metabolites (Fig. S4). Relevant networks enriched by miRNAs included bone marrow cancer (*p* = 0.003), dilated cardiomyopathy (*p* = 5.3 × 10^−6^) and fibrosis of heart (*p* = 3.5 × 10^−7^). In addition, the blood pressure (*p* = 0.003) and coronary disease networks (*p* = 0.04) were also co-enriched by the metabolites linked to cardiovascular disorder.

Figure S5A depicts the alterations between miRNA and metabolites at three different time points for immune related networks. We identified 64 miRNAs and 17 metabolites linked to inflammation. Interestingly, a majority of these molecules, namely, 44 miRNAs and 9 metabolites, were upregulated during the Early phase. A hierarchically clustering of the miRNAs and metabolites that are exclusively linked to immune functions is presented in Fig. S5B.

## Discussion

Curating microRNAs as radiation markers has been gaining significant traction in recent years^13,34–36^. The small endogenous RNA theoretically modulates up to 30% of the entire human genome and thereby regulates a wide variety of cellular and molecular functions. Furthermore, the miRNA-mediated epigenetic regulations can sustainably influence the bio-functional landscape. miRNA expressions and biogenesis have been posited to maintain a bi-directional relationship to metabolic stimuli, such as hormones and endocrines^37^. A shock in the metabolic stimuli influences the miRNA expressions; while the altered miRNA profile can disturb the metabolic homeostatis via epigenetic modulations and transcriptional changes^37^. Therefore, it is clear that metabolites, as the end product of metabolic function, are distinctively connected to miRNA expression. Taken this bi-directional relationship between miRNA-metabolite in consideration, we postulate that a systems integration of serum proteins, miRNAs and metabolites can shed light on the temporal impacts of a lethal dose of radiation on underlying the biofunctions and disease onset.

The clustering trend in the PCA of the global miRNA and metabolite landscape suggested a time lag between the shift in global miRNA expressions and the onset of perturbation in metabolite landscape. The degree of divergence from the baseline miRNA expressions increased gradually with the time lag from TBI. Baseline controls clustered together with Early phase specimens, while Late phase samples (d9 post-TBI) stayed furthest apart from the baseline. In contrast, the metabolite landscape divulged an early separation from the baseline, suggesting an immediate impact of lethal radiation on the metabolic framework.

Taking cues from the sample distribution in the miRNA PCA plot, we segmented the post-TBI time scale into three phases, Early, Mid and Late, respectively. The majority of the miRNA-enriched networks (100 out of 141 networks) emerged significantly perturbed during the Early phase post-TBI and remained significantly enriched across the entire post-TBI time period. Arguably, this trend supports the fact that miRNA mediates an epigenetic mechanism that marks the lasting impacts on biofunctions. Contrastingly, Mid phase post-TBI contained the largest number of metabolite-enriched networks. Unlike the miRNA-enriched networks, only a few of metabolite-enriched networks (18.6% of all 70 networks) remained perturbed through the entire post-TBI time period.

Despite certain differences between miRNA and metabolite-enriched networks, a concerted perturbation of miRNA and metabolite landscape emerged from the systems analysis. miRNA-metabolite integrative analysis suggested an early onset of cardiac atrophy, bone marrow aplasia, and cancer. Tumorigenesis and oncogenesis were linked to 70% of those networks, which were consistently enriched across the entire post-TBI time period. All of the top five highly enriched networks and most of the highly significant miRNAs are linked to various types of carcinoma, a major comorbidity of lethal TBI^38^.

The hematopoietic system is highly susceptible to radiation, and effective countermeasure development is still elusive despite the decades long investigation^39^. Radiation triggers apoptosis in the bone marrow cells, a major site of hematopoiesis^40^. Hence the early enrichment of the bone marrow cancer network was of particular interest. Early accumulation of FLT-3L protein in serum indicated potential perturbation in the network linked to the development of hematopoietic progenitor cells. Overexpressed FLT-3L has been reported in a wide number of hematologic cancers^41,42^; however, several attempts at inhibiting FLT-3L failed to control the malignancies, which essentially raised the speculation about the presence of additional independent factors involved in modulating the progenitor cell growth^43^. Our study identified a number of miRNAs with known involvement in regulating the differentiation of hematopoietic cells under lethal radiation. A major subset of these miRNAs are associated with NFkB signal, a known regulator of hematopoietic cell differentiation^44^. For instance, we reported an early escalation of miR-155–5p, which has been found to be co-expressed with NFkB signals in malignant cells; otherwise miR-155-5p maintains at low-level expression in mature hematopoietic cells^45^. In concurrence, we reported a set of inhibited miRNAs, such as miR-486-5p, miR-21-5p and miR-351-5p that are known to regulate hematopoiesis via NFkB signal^46,47^.

As a potential promoter of survival mechanisms, the activation of EPO is typically triggered by hypoxia and low hematocrit^48,49^. Highlighting the relevance of EPO in responding to radiation, several independent studies reported elevated EPO as the consequence of both lethal and sublethal radiation doses^18,50^. In concurrence, we found a gradual increment of EPO load that reached significant levels at d7 post-TBI. As an established modulator of cancer^51^ miR-125b-5p is also an upstream regulator of EPO^52^; the inhibited signal of miR-125-5p during the Early phase was probably linked to the continued increase of EPO load during the Mid to Late phases post-TBI. Furthermore, activated EPO is an established upstream regulator of miR-486-5p^53^ and miR-451a^54^. Together this molecular axis comprised of mir-125b-5p → EPO → miR-486-5p and miR-451a could be of great importance in the context of erythropoiesis caused by lethal radiation dose.

Radiation-induced cardio atrophy, otherwise known as radiation cardiotoxicity, is of noteworthy clinical interest. With the present treatment regimen, cardiotoxicity typically manifests as a delayed effect of radiation^55^. However, in the present study exposing mice to a lethal dose of radiation, the compromised heart and vascular system emerged as an acute health issue. Our study suggested inhibition of serum fatty acids, which are often identified as the integral part of the protective mechanism against of cardiotoxicity^56^. In addition, we identified a number of miRNAs that have been linked to cardiomyopathy^57,58^. For instance, miR-30c-5p, miR-126a-5p, miR-378, miR-16-5p, miR-140-3p, miR-19b-3p, miR-199a-3p and miR-17-5p were linked to the cardiovascular atrophy caused by a lethal dose of radiation. According to the literature, all of these miRNAs have been linked to cardiac diseases caused by non-radiation insults^58^. A recent study exploring the cardiac atrophy caused by lethal radiation underscored the key role of the TGF-beta signal in heart fibrosis, metabolic disordering, and impaired contractility^59^. Supporting this evidence we found two TGF-beta-associated molecules, namely, miR-17-5p^60^ and sildenafil^61^, differentially expressed during the Early phase post-TBI.

Release of ROS is a typical comorbidity of radiation, a phenomenon that is regularly observed among patients undergoing radiotherapy^62^. In agreement, we found a significant enrichment of the ROS synthesis network in irradiated mouse plasma. A set of fatty acids including linoleic acid, docosahexaenoic acid, octadecadienoic acid, docosahexaenoic acid, and palmitic acid became inhibited as the immediate response of TBI, which could be attributed to that fact that fatty acid metabolism is a key source of ROS biogenesis^63^. It also important to note that the release of ROS is a key signature of cardiovascular disorder^64^ and bone marrow aplasia^65^.

An early surge of inflammation is the hallmark signature of a host response to radiation^66^. Our miRNA-metabolite integrative analysis found a consistent perturbation of inflammatory signals across the time line. A number of studies have diligently established miRNAs as a strategic check point of host immunity, in particular the innate immunity^67^. Via transcriptional alteration of the immunomodulating factors, miRNAs can act as pro-inflammatory or anti-inflammatory agents^68^. For instance, miR-155 can act in a dual role accomplishing both anti- and pro-inflammatory functions, and our study reported an elevated serum miR-155-5p during the Early phase post-TBI. The potential synergistic role of miR-155-5p and the NFkB network in hematopoiesis^69^ could be exploited further to develop a next generation therapeutic strategy against radiation. miR-124-3p is another anti-inflammatory candidate of interest that was consistently inhibited across all time phases post-TBI. This miRNA also directly targets NFkB, and its downregulated form is known to elicit an inflammatory response. In addition, we identified several other pro-inflammatory (miR-125-5p and miR-23a-3p)^70^ and anti-inflammatory (miR-124-3p, miR-145-5p, miR-181a-5p, miR-21-5p and miR-223-3p)^68^ miRNA signatures of potential translational values. Furthermore, this inflammatory response was likely causally related to the early comorbidities of lethal radiation, namely, bone marrow aplasia^71^, cardiovascular disease^72^ and ROS synthesis^73^, as discussed earlier.

In conclusion, the investigation of the lethal radiation’s temporal influence on serum miRNAs and metabolites curated time critical markers and relevant molecular mechanisms triggered by TBI. At present, we precluded the analysis of the whole genome mRNA profile despite the fact that mRNA readouts could be the logical bridge between miRNA and metabolite readouts. Instead, we focused on the molecules available in the blood serum. We validated the longitudinal loads of three proteins of interest and built non-canonical networks around these proteins to understand the molecular etiology. Inflammation emerged as an overarching host response, which was potentially associated with some of the early disease footprints, such as dysregulated hematopoiesis and cardiovascular disorder. In future studies, we will embark on validating the translational potential of these markers using animal models from a higher phylogenetic order, as well as identifying differences in the sexes.

## Supplementary information

Supplementary Information.
