# Supplementary material for: microRNA and Metabolite Signatures Linked to Early Consequences of Lethal Radiation
Source: Sci Rep. 2020 Mar 25;10:5424. doi: 10.1038/s41598-020-62255-w (PMC7096415; doi:10.1038/s41598-020-62255-w)

*micro*RNA and Metabolite Signatures Linked to Early Consequences of Lethal Radiation

Nabarun Chakraborty[1,2], Aarti Gautam[2], Gregory P. Holmes-Hampton[5], Vidya P. Kumar[5], Shukla Biswas[5], Raina Kumar[3], Dana Hamad[4], George Dimitrov[1,2], Ayodele O. Olabisi[5], Rasha Hammamieh[2], and Sanchita P. Ghosh[5]

[1]The Geneva Foundation, US Army Center for Environmental Health Research, Fort Detrick, MD, 21702-5010, [2]US Army Center for Environmental Health Research, Fort Detrick, MD, 21702-5010, [3]ABCC, Fredrick National Lab for Cancer Research, Fort Detrick, MD, 21702-5010, [4]ORISE, US Army Center for Environmental Health Research, Fort Detrick, MD, 21702-5010, [5]Armed Forces Radiobiology Research Institute, Uniformed Services University of the Health Sciences, Bethesda, MD 20889

Supplementary Figure S1

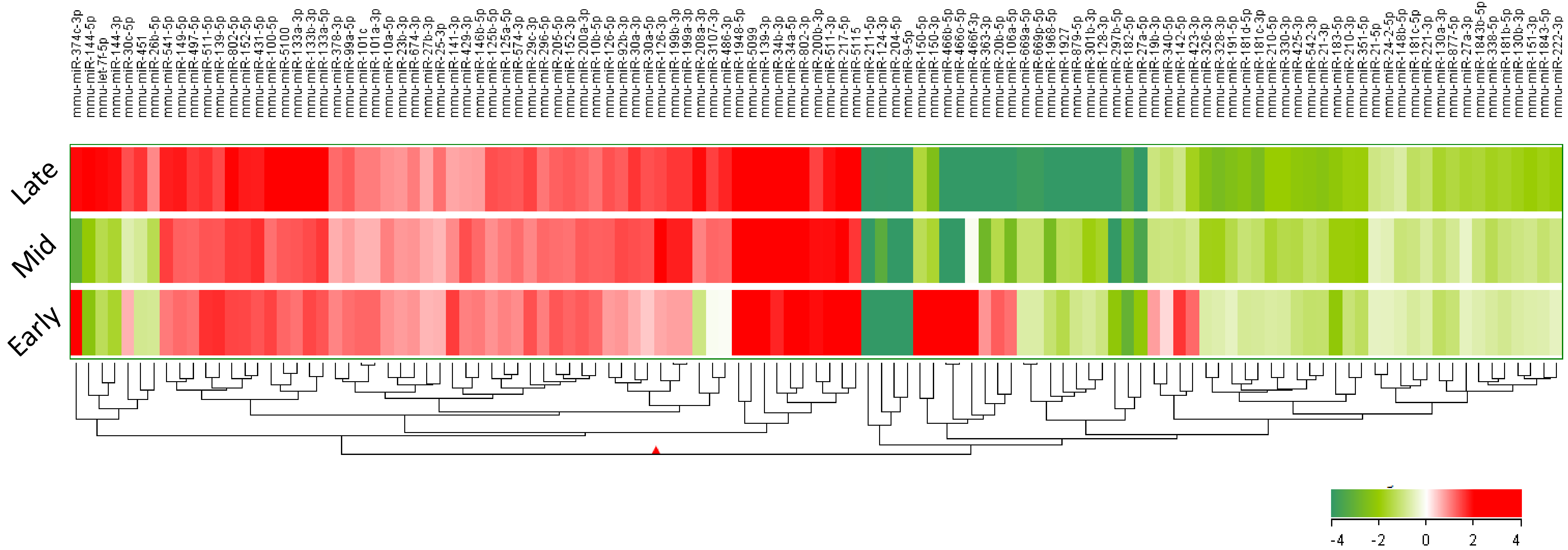

Supplementary Figure S2.

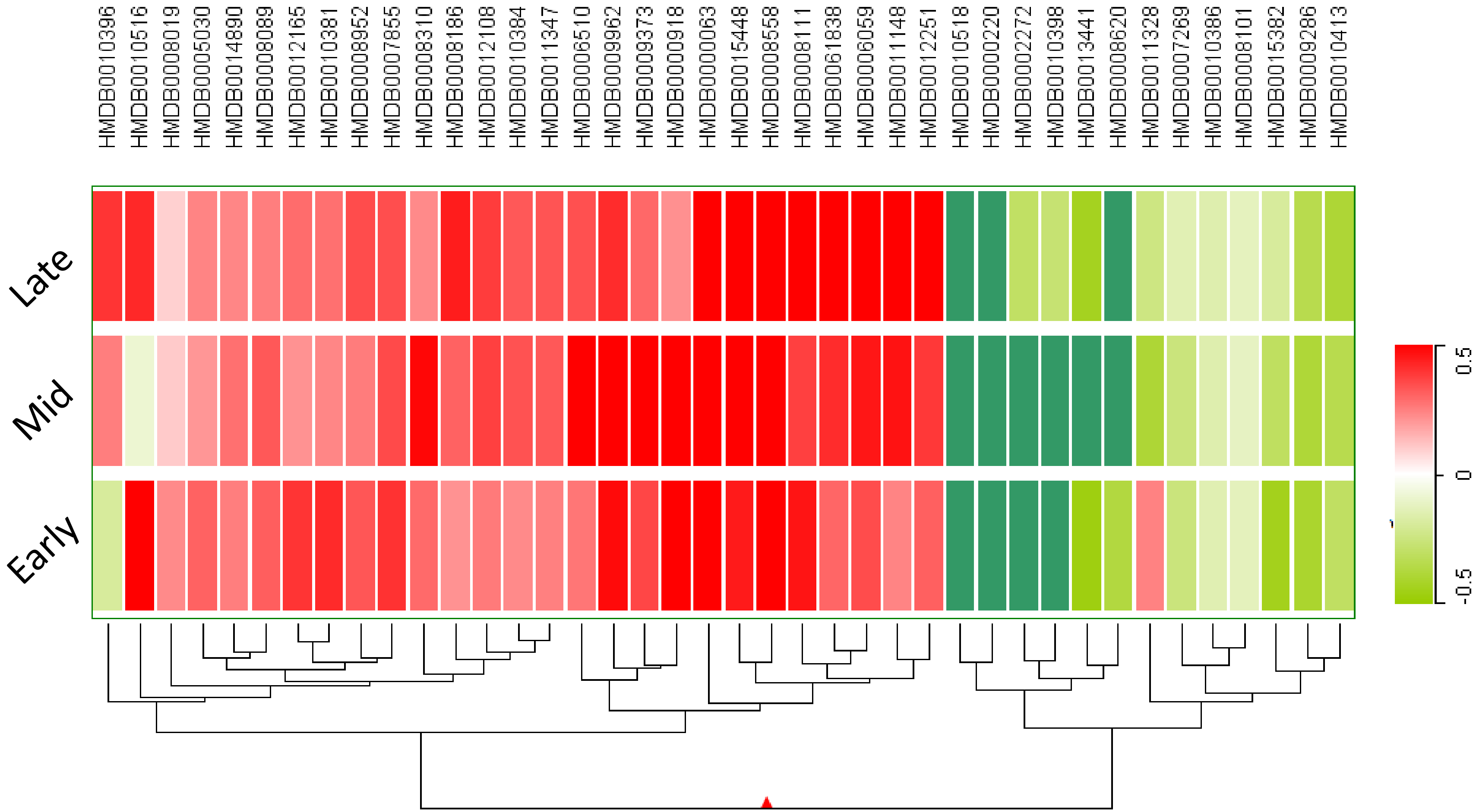

Supplementary Figure S3.

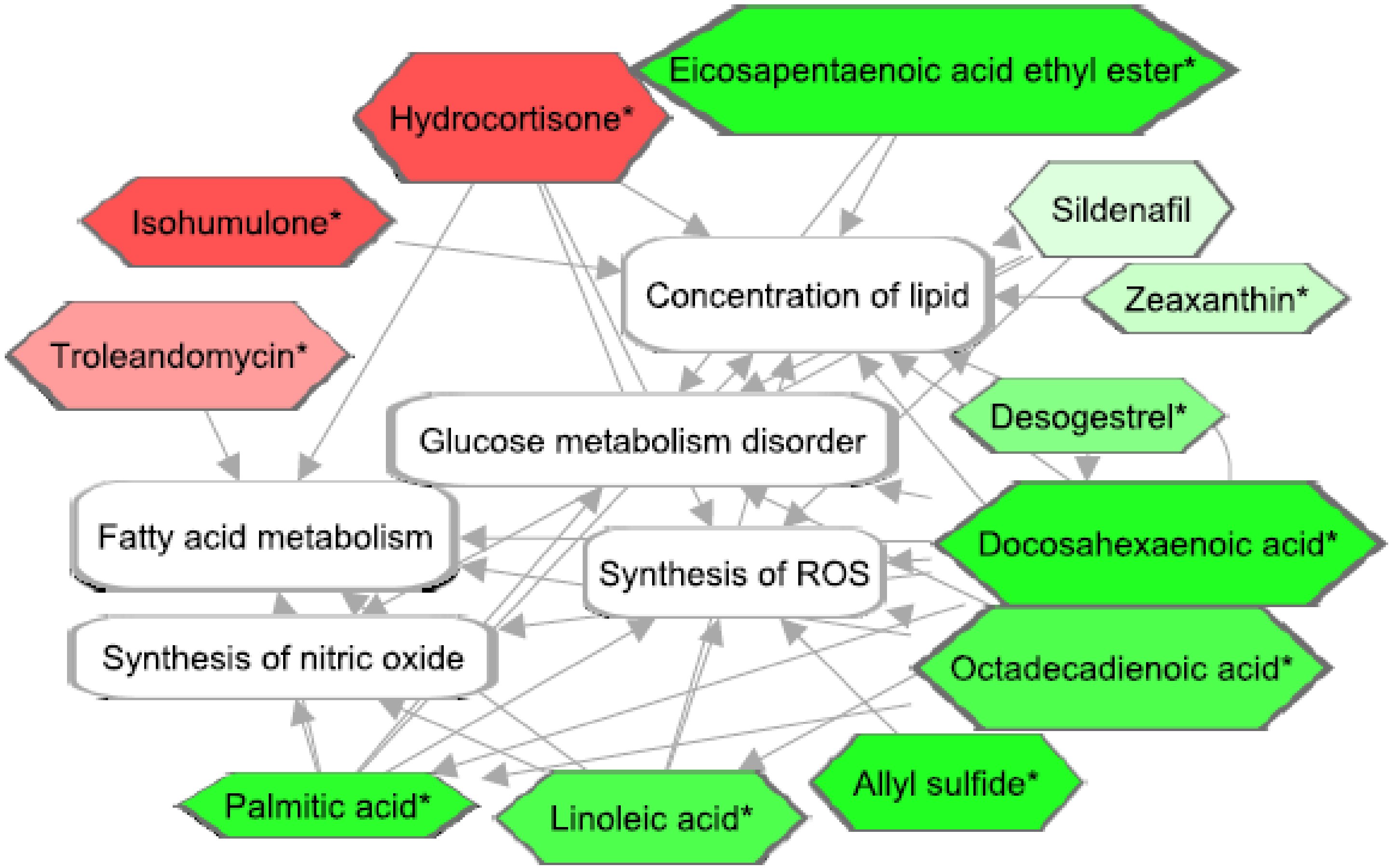

Supplementary Figure S4.

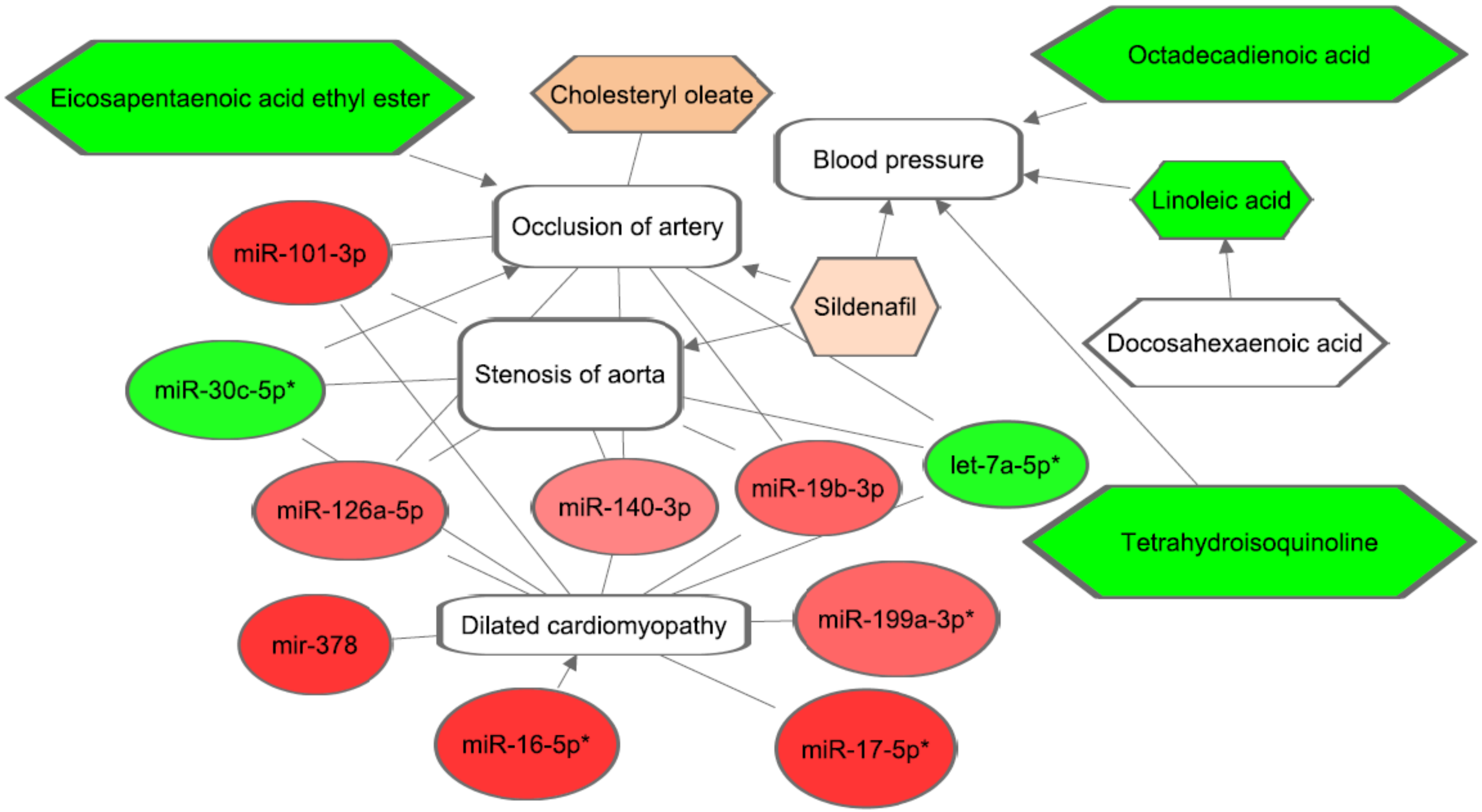

Supplementary Figure S5A.

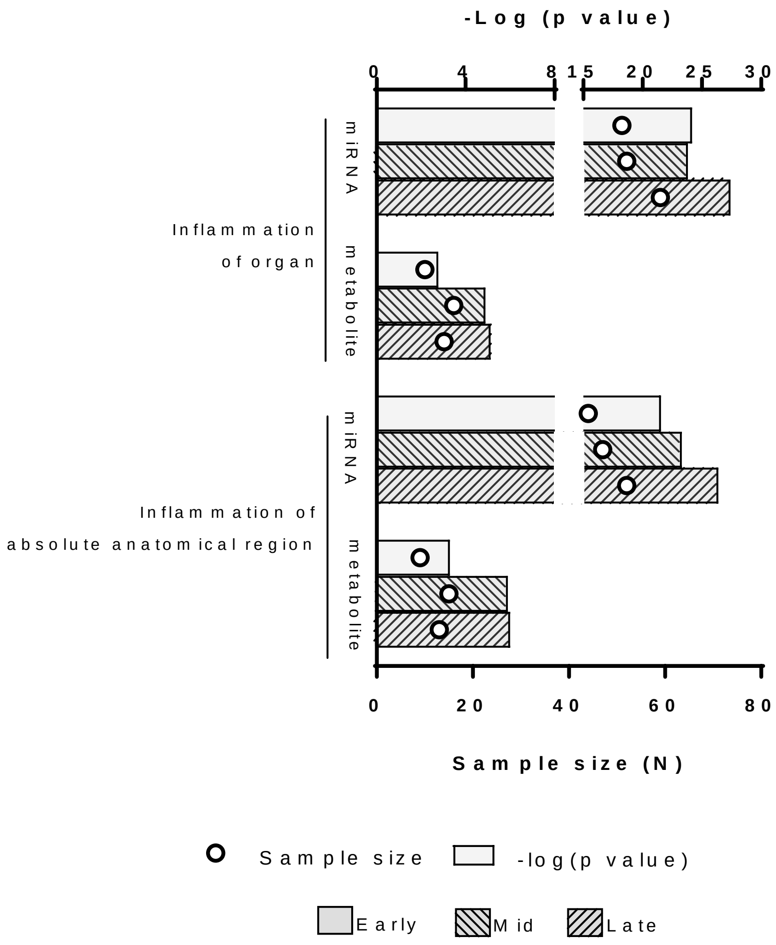

Supplementary Figure S5B.

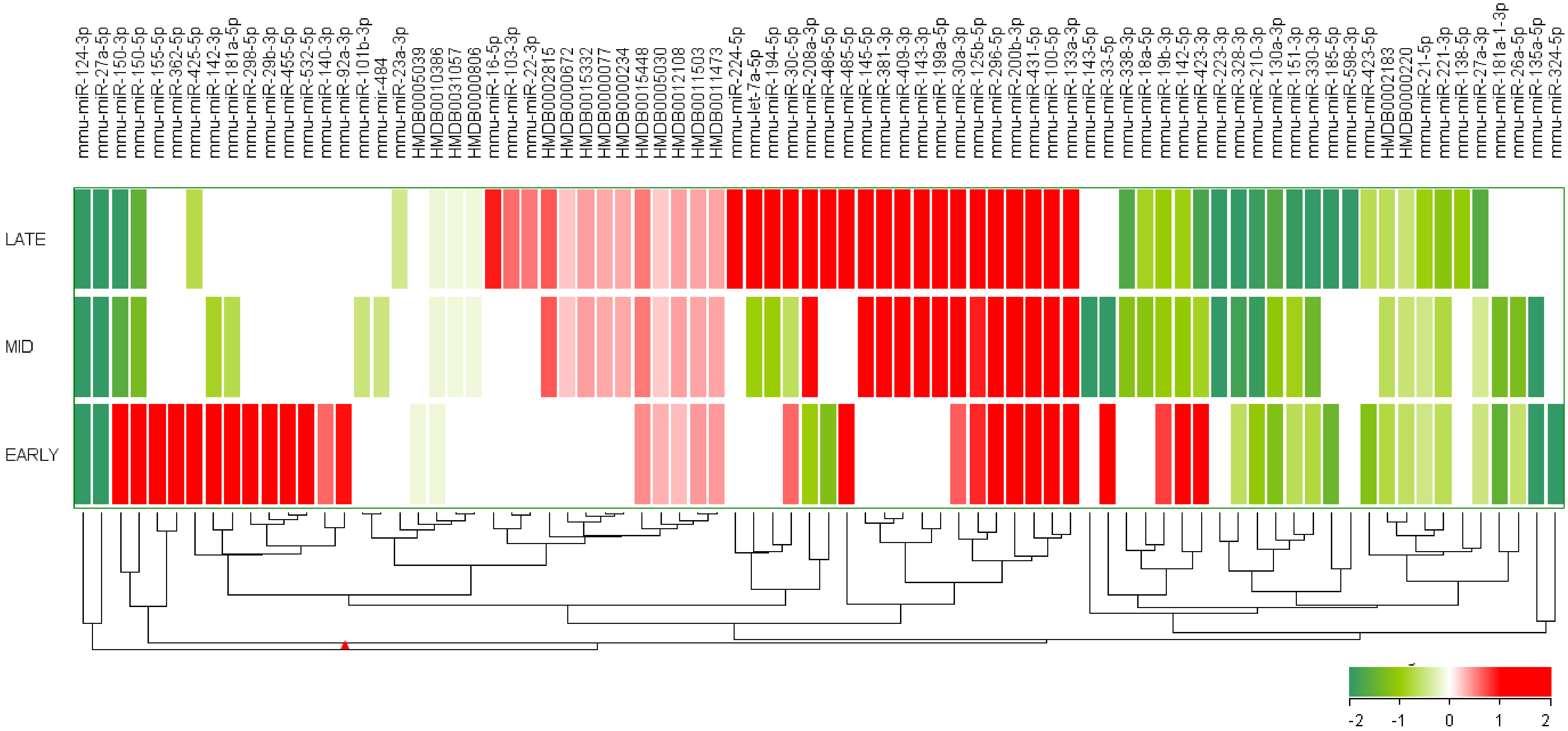

Supplement: Supplementary file 1 — Supplementary Information. [file 41598_2020_62255_MOESM1_ESM.pdf]
